# Comparing Intra-articular Platelet-Rich Plasma With Hyaluronic Acid for the Treatment of Hip Osteoarthritis: A Systematic Review and Meta-Analysis

**DOI:** 10.7759/cureus.47919

**Published:** 2023-10-29

**Authors:** Hembashima G Sambe, Mohamed Yasir, Ruzhual K Man, Amaresh Gogikar, Ankita Nanda, Lakshmi Sai Niharika Janga, Pousette Hamid

**Affiliations:** 1 Surgery, California Institute of Behavioral Neurosciences and Psychology, Fairfield, USA; 2 Internal Medicine, California Institute of Behavioral Neurosciences and Psychology, Fairfield, USA; 3 Medicine, California Institute of Behavioral Neurosciences and Psychology, Fairfield, USA; 4 Neurology, California Institute of Behavioral Neurosciences and Psychology, Fairfield, USA

**Keywords:** intra-articular injection, hyaluronic acid, platelet-rich plasma (prp), osteoarthritis (oa), hip

## Abstract

Hip osteoarthritis (HOA), a prevalent condition among those aged 55 years and above, is a significant cause of joint pain and functional impairment and it contributes to the overall burden of chronic pain experienced by the elderly population. While platelet-rich plasma (PRP) and hyaluronic acid (HA) injections have emerged as innovative therapeutic approaches for managing osteoarthritis, their effectiveness in HOA remains a subject of contention. Therefore, the objective of this review was to assess the efficacy of PRP versus HA in terms of pain relief and functional outcomes for the management of HOA. We searched PubMed, Cochrane Library, and Google Scholar databases from 2013 to 2023 to identify pertinent randomized controlled trials (RCTs). A total of seven trials (478 participants) were included. The selected studies underwent quality assessment using the updated Cochrane risk of bias tool. The pain and functional outcomes were examined using measures of the Western Ontario and McMaster Universities Osteoarthritis Index (WOMAC) pain scale, visual analog scale (VAS), and Harris hip score (HHS). In the meta-analysis, standard mean differences (SMDs) along with 95% confidence intervals (CIs) to estimate overall effect magnitudes for continuous outcomes were extracted. Statistical significance was determined using p-values below 0.05. At six months, the PRP group experienced a significantly lower standard mean WOMAC pain score (SMD = -0.38, CI = -0.64 - 0.13; p = 0.03). No significant differences in WOMAC pain scores were noted at one to two months (SMD = 0.09, CI = -0.24, 0.43; p = 0.59), and at 12 months (SMD = -0.85, CI = -1.81, 0.12; p = 0.09). Similarly, for VAS, patients on PRP showed a slight improvement in their VAS scores at six months (SMD = -0.50, CI = -0.89, -0.12; p < 0.01). However, no significant differences in VAS between the PRP groups and the HA groups were observed at one to two months (SMD = -0.22, CI = -0.49, 0.04; p = 0.10) and at 12 months (SMD = -0.22, CI: -0.63, 0.19; p = 0.29). In terms of hip dysfunction, there was no statistically significant standard mean difference in HHS between the PRP and HA groups at six months (SMD = 0.02, CI = -0.40, 0.44; p = 0.93), and at 12 months (SMD = -0.31, CI = -0.32, 0.22; p = 0.73). This review and meta-analysis provide insights into emerging treatments for HOA, especially considering that PRP shows potential benefits and safety for patients with HOA during mid-term follow-up in a 12-month period. Nevertheless, it is necessary to conduct research that includes high-quality designs and larger sample sizes to validate the comparative efficacy of these treatments.

## Introduction and background

Osteoarthritis (OA) is a common degenerative disorder characterized by joint pain, stiffness, and functional limitations. In the context of hip osteoarthritis (HOA), there is gradual femoroacetabular cartilage loss, leading to inflammation, narrowing of the hip joint space, periarticular ligament laxity, and muscle weakness [1]. OA also leads to sub-chondral bone alterations, osteophyte formation, and synovial hyperplasia [2]. The loss of structural integrity of cartilage lining the hip’s articular surface arises from the degradation of the following two major components within the extracellular matrix of articular cartilage: aggrecan and collagen. Aggrecan molecules are degraded by specific enzymes having aggrecanase activity called A disintegrin and metalloproteinase with thrombospondin motifs (ADAMTS). Collagen is degraded by matrix metalloproteinases (MMPs). The increase in the degradation of the articular cartilage matrix and the decrease in its synthesis leads to irreversible destruction of articular cartilage [3]. The incidence of OA is higher in people aged 65 years or above and in females than males. The prevalence is also increasing with time. In the United States only, an estimated 10-25% of people over 60 years of age have HOA and factors like obesity, increased age, cardiovascular diseases, hypertension, diabetes, previous hip joint trauma, and female sex contribute to its development [4].

In today’s medical world, different management strategies are used for OA. Non-surgical interventions include physical therapy, non-steroidal anti-inflammatory drugs (NSAIDs), platelet-rich plasma (PRP), hyaluronic acid (HA), and corticosteroids. Among these treatments, HA is a high molecular weight polysaccharide that is crucial in protecting chondrocytes. It is widely present in many human body tissues, including heart valves, umbilical cord, synovial fluid, skin, and skeleton tissues. With the development of OA, the concentration of HA decreases, resulting in decreased viscoelasticity of synovial fluid and degeneration of articular cartilage. Thus, intra-articular HA injection is used as a standard treatment for OA including HOA [5]. Similarly, PRP is also used to treat a wide range of musculoskeletal diseases. It comprises concentrated platelets, leukocytes, and fibrin. Moreover, through exocytosis, activated platelets release cytokines, transforming growth factors, and other compounds that also aid tissue repair [6]. According to widespread consensus, PRP affects the therapy of OA in the following three different ways: by controlling immunity, limiting inflammatory responses, and regulating cell metabolism through growth factors [7]. Clinical studies indicate that PRP is safe and a promising approach for treating OA [8].

A systematic review conducted in 2022 revealed that while PRP and HA had comparable positive short-term clinical effects in the treatment of HOA, there were no significant differences in the weighted improvement of any outcome score from pre-injection to post-injection between groups [9]. The findings may have been attributable to various PRP preparation techniques, application regimens, and leukocyte concentrations used in the included randomized controlled trials (RCTs). Other studies have also revealed that the quantity of PRP injections impacts the therapy of OA [10]. Owing to the sparseness of studies reporting the comparative efficacy of intra-articular PRP with HA, especially for HOA, these treatments remain a subject of ongoing debate and investigation. Therefore, a comprehensive and critical evaluation of the available evidence is necessary to guide clinical decision-making and optimize patient care in terms of treatment modality for pain reduction, functional improvement, and long-term disease outcomes for HOA.

The objective of this study was to compare the efficacy of PRP with HA in terms of pain and functional outcomes for patients with HOA using available RCTs. We hypothesize that clinical outcomes differ between PRP and HA treatments for patients diagnosed with HOA. By synthesizing existing clinical evidence, this study seeks to provide a comprehensive review of these interventions' relative benefits and limitations, thereby helping clinicians and patients make informed choices based on the most up-to-date information. The review also aimed to evaluate the outcomes of HOA patients based on the adverse effects of treatment with PRP and HA.

## Review

Methods

The Preferred Reporting Items for Systematic Reviews and Meta-Analyses (PRISMA) 2020 guidelines were employed in this systematic review [11]. The population comprised individuals aged 18 years and above diagnosed with HOA and being managed for HOA pain and functionality issues. The intervention was treatment with intra-articular PRP while the comparator was treatment with intra-articular HA. The primary outcome was pain reduction and improvement in hip functional outcomes. The secondary outcome was adverse event profiles of patients on PRP or HA intra-articular injection treatments.

Inclusion Criteria

The review focused on RCTs published in English, comparing the efficacy of intra-articular PRP with HA for HOA over the last decade (2013-2023). Studies evaluating patients with a confirmed diagnosis of HOA, ranging from adults to the elderly, were included in this review. We also included studies highlighting the results of treatment with PRP compared to HA for patients with HOA, aimed at reducing pain and inflammation and increasing the overall quality of life. For the primary outcome, studies using the Western Ontario and McMaster Universities Index (WOMAC) and Visual Analog Scale (VAS) to evaluate pain and studies using the Harris hip score (HHS) to evaluate hip functional outcomes were considered [12-15].

Exclusion Criteria

The studies falling within the domains of case series, quasi-randomized controlled trials (quasi-RCTs), case reports, non-randomized controlled trials (non-RCTs), ongoing trials, in vitro and in vivo studies, studies lacking complete data, studies containing inaccuracies as well as studies employing incorrect intervention methodologies were not considered. Additionally, studies focused on hip joint diseases like rheumatoid arthritis and gout were excluded as well. Studies evaluating patients with joint replacement and prostheses were also not included in the study.

Search Strategy

The search strategy focused on identifying RCTs from 2013 to 2023 based on predefined inclusion and exclusion criteria. In the initial search stage, relevant keywords were identified through simple synonym searching and extracted from previously published relevant papers. These keywords were then used to search databases, and cross-referencing was conducted to identify commonly used MeSH terms that aligned with the inclusion criteria. The reviewers employed the snowball method to scrutinize records and select all studies eligible for retrieval and final screening for potential inclusion. The search results were restricted based on the criteria of each database by utilizing relevant search filters as shown in Table 1.

**Table 1:** Databases used for collecting articles with search strategies and appropriate filters. MeSH: Medical Subject Headings; PRP: platelet-rich plasma

Database used	Search strategy	Number of research articles retrieved
PubMed	({({({["platelet-rich plasma" [All Fields] OR "platelet-rich plasma" [All Fields] OR "prp" [All Fields])} AND ("hyaluronic acid" [Supplementary Concept] OR "hyaluronic acid" [All Fields] OR "hyaluronic acid" [MeSH Terms] OR {"hyaluronic" [All Fields] AND "acid" [All Fields]})) OR ("hyaluronate" [All Fields] OR "hyaluronates" [All Fields] OR "hyaluronic" [All Fields])} AND ("osteoarthritis" [MeSH Terms] OR "osteoarthritis" [All Fields] OR "osteoarthritides" [All Fields]) OR ("arthritis" [MeSH Terms] OR "arthritis" [All Fields] OR "arthritides" [All Fields] OR "polyarthritides" [All Fields]) AND ("hip" [MeSH Terms] OR "hip" [All Fields])] OR ("hip joint" [MeSH Terms] OR ("hip" [All Fields] AND "joint" [All Fields]) OR "hip joint" [All Fields]) AND (y_10 [Filter]) AND (randomized controlled trial [Filter]})	1290
Google Scholar	Allintitle: platelet-rich plasma hyaluronic acid hip osteoarthritis	20
Cochrane Library	Platelet-rich plasma AND hyaluronic acid AND hip osteoarthritis	20
	PRP AND hyaluronic acid AND hip osteoarthritis	17

Study Selection

The selection process involved the removal of duplicates and reviewing titles and abstracts to identify full-text articles that would be eligible for retrieval and screening. The selected full-text articles were carefully screened to identify articles relevant for inclusion. The finalized papers were checked for quality and potential bias prior to analysis.

Risk of Bias Assessment

Two review authors assessed the quality and risk of bias in each study by using the revised Cochrane risk of bias (RoB2) tool [16]. The examination domains included biases arising from the randomization process, deviations from intended interventions, missing outcome data, measurement of the outcome, and selection of the reported result [16]. After responding to the signaling questions, one of three types of bias judgments was selected namely “low,” “high,” and “some concerns.” In the case of conflicts, a third author was contacted as an unbiased arbitrator.

Statistical Analysis

The meta-analysis was conducted using the RevMan Web software version: 6.4.1 (London, UK: The Cochrane Collaboration) [17]. For this purpose, the WOMAC-pain, VAS, and HHS function scores were regarded as continuous in nature and the random effects model and inverse variance approach for continuous outcomes were utilized [18]. We used the WOMAC pain scale which is one of three scales comprising the WOMAC index. The WOMAC pain scale addresses five items that evaluate pain with walking, stair climbing, sitting, lying down, and standing; higher WOMAC scores indicate a worse outcome [12]. Similarly, a higher VAS score indicates poorer outcomes [13,14]. However, for the HHS which measures hip dysfunction, a lower score indicates poorer outcomes [15].

The standard mean differences (SMDs) between the treatment groups, along with the 95% confidence intervals (CIs) were used to estimate the overall effect magnitudes for continuous outcomes [18]. This was achieved by extracting the means and standard deviations (SD) of pain and function scores from the studies included. When mean values were available during data extraction but SD was missing, the RevMan Calculator tool computed the SD using the mean, 95% CI, and sample size [18,19]. Statistical significance was determined using p < 0.05. In evaluating the extent to which the observed variation across experiments could not be attributed to random chance, we employed the I^2^ statistic and conducted a Chi-square test (p < 0.05). In our analysis, a p < 0.05 and an I^2^ value below 50% were indicative of low heterogeneity. Sub-analyses were performed for any outcome score in which more than five studies reported results.

Results

Study Selection and Quality Evaluation

**Figure 1:**
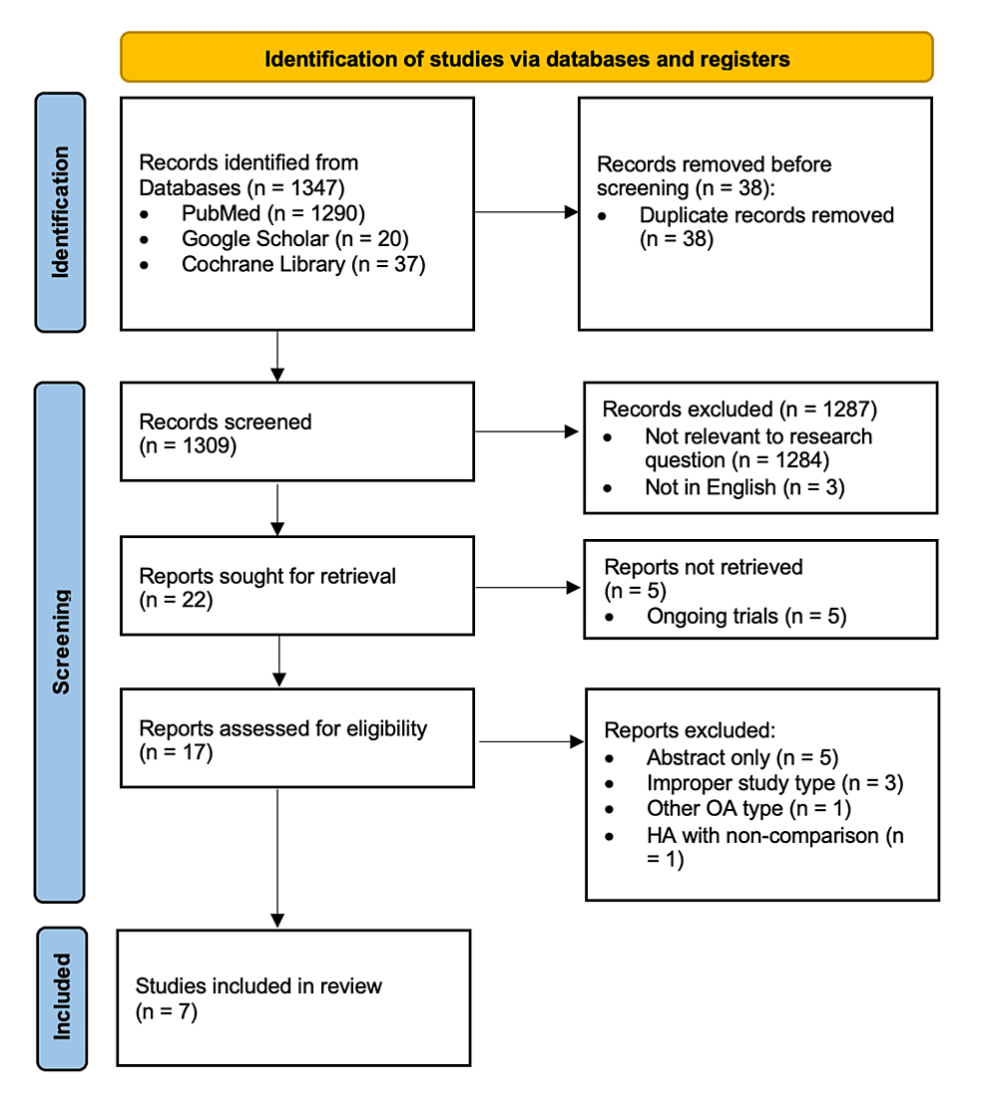
PRISMA flowchart showing the search, screening, and inclusion process. PRISMA: Preferred Reporting Items for Systematic Reviews and Meta-Analyses

From the period between 2013 and 2023, a comprehensive set of 1347 studies was identified across the following databases: PubMed, Google Scholar, and Cochrane Library. After removing 38 duplicate entries, 1309 unique records were screened. Out of 22 potentially relevant studies identified through screening, 17 records were retrieved and assessed for final eligibility and inclusion. Seven studies were deemed suitable for inclusion in the systematic review and subsequent meta-analysis. The PRISMA diagram in Figure 1 outlines the entire study selection process and the reasons for exclusion [11].

Study Characteristics

Studies that were incorporated in this analysis were conducted in Italy, Iran, the United States, and Spain [20-26]. A total of 478 participants, aged 18 years and above, and diagnosed with HOA were included in a collection of seven studies. In all seven studies, HA was used as a comparator to PRP. Two studies reported low leukocyte PRP [24,26]. The WOMAC score was recorded by five studies [22-26]. Of the six studies that measured VAS, four of them used a 10-point VAS to measure pain [20,22,25,26]. The other two studies used a 100-point VAS [21,23]. Three studies recorded the HHS [20,23,26]. Table 2 shows a detailed summary of studies that were included in the review.

**Table 2:** Study characteristics of the included studies. HA: hyaluronic acid; HHS: Harris hip score; HMW: high molecular weight; Lp PRP: leukocyte poor PRP; LMW: low molecular weight; Na: sodium; NR: not reported; OARSI: Osteoarthritis Research Society International; PRP: platelet-rich plasma; RCT: randomized controlled trial; VAS: visual analog scale; WOMAC: Western Ontario and McMaster Universities Arthritis Index

Authors	Country	Study design	Number of participants	Average age (years)	Intervention	Duration	Outcomes measured
Dallari et al. [21]	Italy	RCT	80 (PRP = 44, HA = 36)	NR	Autologous PRP (5 mL) versus HA (30 mg/2 mL)	12 months	Therapeutic efficacy of autologous PRP; therapeutic efficacy of HA
Di Sante et al. [22]	Italy	RCT	43 (PRP = 21, HA = 22)	PRP = 71.37±6.03, HA = 73.62±7.87	Na-HA (30 mg/2 mL) versus PRP (3 mL)	16 weeks	Pain reduction was measured by VAS; pain reduction was measured by WOMAC pain scale
Kraeutler et al. [24]	USA	Double-blind, randomized pilot study	31 (PRP = 18, HA = 13)	PRP = 53.3±8.4, HA = 53.6±7.6	Lp PRP (1-2 mL) versus LMW HA (2.5 mL)	12 weeks	Efficacy of intra-articular injection of Lp PRP; efficacy of intra-articular injections of LMW HA
Villanova-López et al. [26]	Spain	Phase III double-blinded, controlled trial	74 (PRP = 38, HA = 36)	PRP = 61.2±9.72, HA = 61.1±12.3	PRP (6 mL) versus HA (60 mg/6 mL)	12 months	Pain was assessed using VAS score; HHS score was used as functional score; WOMAC score was used as functional score; analgesia, adverse events, cellular components in peripheral blood, cellular components in PRP, and clinical response were assessed using OARSI criteria
Nouri et al. [25]	Iran	RCT with three parallel groups	70 (PRP = 35, HA = 35)	PRP = 58.22±5.10, HA = 60.93±4.54	PRP 5 mL versus HMW HA 50 mg/2.5 mL	Two injections within two weeks' interval	VAS; WOMAC; Lequesne questionnaire
Doria et al. [23]	Italy	Prospective double-blinded RCT	80 (PRP = 40, HA = 40)	PRP = 67.3±5.8, HA = 68±4.6	PRP 5 mL versus HA (15 mg/mL)	12 weeks	VAS; WOMAC
Battaglia et al. [20]	Italy	RCT	100 (PRP = 50, HA = 50)	PRP = 51±12, HA = 56±12	Autologous PRP (5 mL) versus HMW HA (30 mg/2 mL)	12 months	HHS; VAS

Risk of Bias Assessment

**Figure 2:**
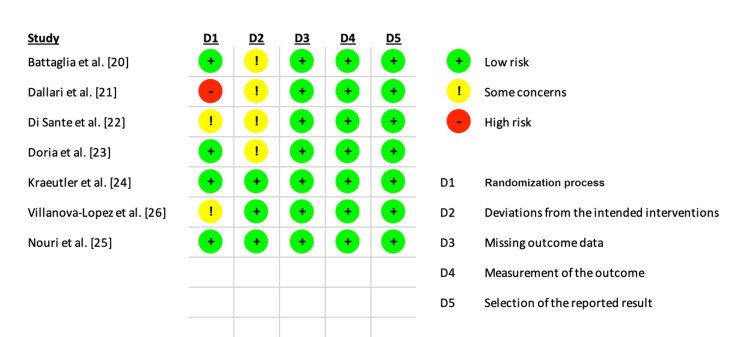
Risk of bias results for the included studies.

**Figure 3:**
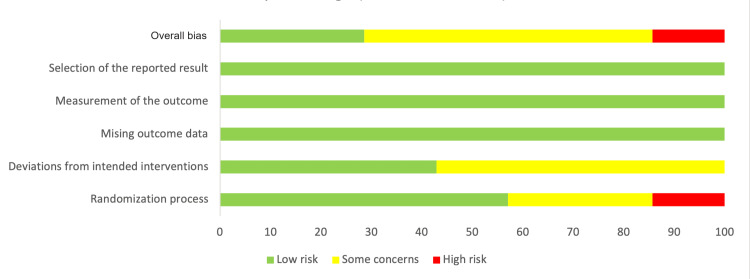
Risk of bias summary chart for the included studies.

The RoB 2 adjudication process shown in Figures 2, 3 demonstrated some concerns with the randomization process and participant blinding. However, there were no perceived factors affecting the outcome evaluations and reporting of results.

Results of Meta-Analysis

Pain assessment (WOMAC pain scale): A total of five studies were assessed for standard mean differences between PRP and HA [22-26]. Four of the studies were analyzed at one to two months [22,24-26]. At the six-month time point, four studies were assessed [23-26]. Only two studies were analyzed at the 12-month follow-up period [23,26].

**Figure 4:**
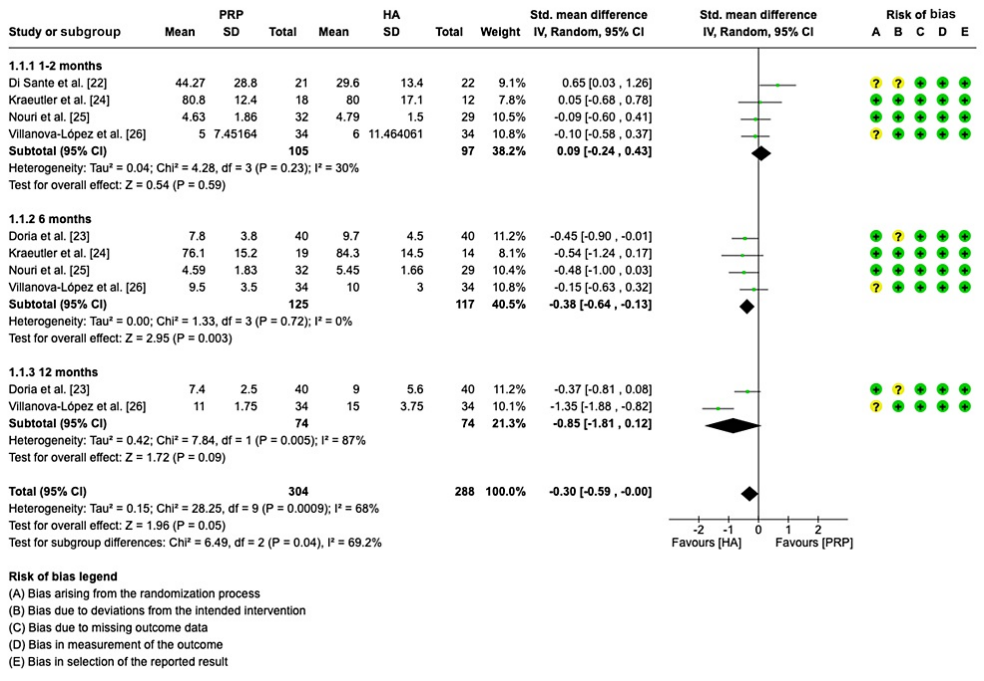
Forest plot showing standard mean differences in WOMAC pain for PRP versus HA. WOMAC: Western Ontario and McMaster Universities Osteoarthritis Index; PRP: platelet-rich plasma; HA: hyaluronic acid; IV: inverse variance

The pooled forest plot with WOMAC-pain as an outcome is shown in Figure 4. Forest plot data analysis indicates that patients on HA experienced higher overall standard mean WOMAC-pain in all subgroups (SMD = -0.30, CI = -0.59, -0.00, p = 0.05; I^2^ = 68%, p = 0.0009). The forest plot did not show statistically significant differences in pain between the PRP group and the HA group at one to two months (SMD = 0.09, CI: -0.24, 0.43, p = 0.59; I^2^ = 30%, p = 0.23), and 12 months (SMD = -0.85, CI: -1.81, 0.12, p = 0.09; I^2^ = 87%, p = 0.005). However, at six months, the HA group experienced a 0.38 higher standard mean WOMAC pain score (SMD = -0.38, CI: -0.64 - 0.13; p = 0.03; I^2^ = 0%, p = 0.72).

Pain assessment (VAS): In the meta-analysis to evaluate the standard mean differences of VAS between the PRP group and HA group, a total of six studies were included [20-23,25,26]. Five studies were assessed in the one to two-month time-point analysis [20-22,25,26]. Similarly, five studies were included in the six-month analysis [20,21,23,25,26]. Four studies were assessed at the 12-month follow-up period [20,21,23,26].

**Figure 5:**
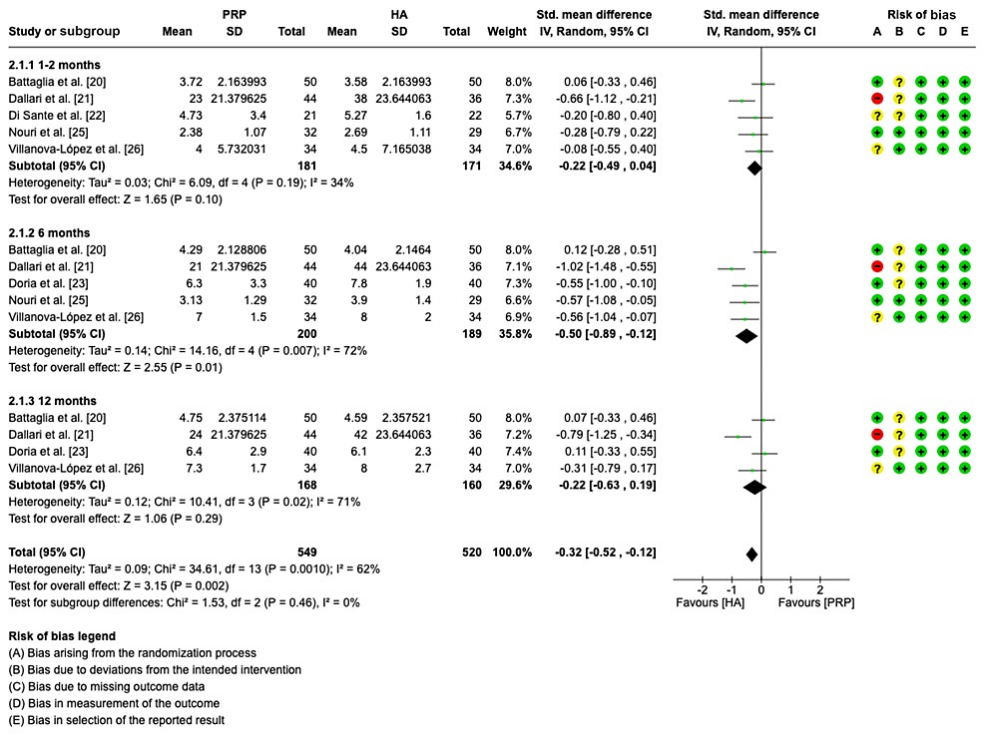
Forest plot showing standard mean differences in VAS for PRP versus HA. VAS: visual analog scale; PRP: platelet-rich plasma; HA: hyaluronic acid; IV: inverse variance

The pooled forest plot findings shown in Figure 5 reveal that PRP significantly improved overall VAS in all subgroups (SMD = -0.32, CI= -0.52, -0.12, p < 0.002; I^2^ = 62%, p = 0.0010). The forest plot further showed a significantly lower VAS standard mean score in the PRP group versus the HA group at six months. However, no significant differences in VAS between the PRP groups and the HA groups were found at one to two months (SMD = -0.22, CI: -0.49, 0.04, p = 0.10; I^2^ = 34%, p = 0.19) and 12 months (SMD = -0.22, CI: -0.63, 0.19; p = 0.29; I^2^ = 72%, p = 0.02).

Functional assessment (HHS): Three studies were included in the meta-analysis to evaluate the standard mean differences of HHS between PRP group and HA group at six months [20,23,26]. At the 12-month follow-up, two studies were included [20,23].

**Figure 6:**
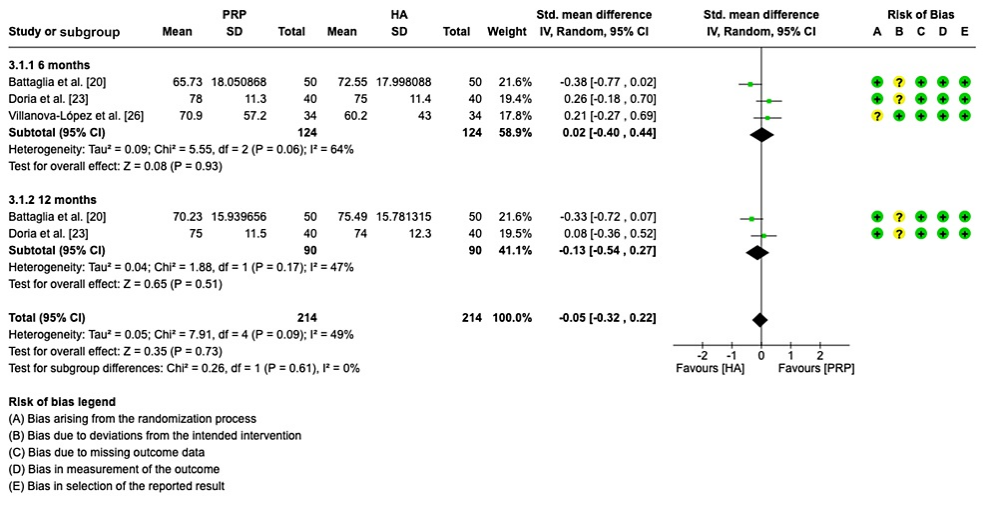
Forest plot showing standard mean differences in HHS for PRP versus HA. HHS: Harris hip score; PRP: platelet-rich plasma; HA: hyaluronic acid; IV: inverse variance

The pooled forest plot findings in Figure 6 indicate that there was no significant difference in overall HHS between PRP and HA in all subgroups (SMD = - 0.05, CI = -0.32, 0.22, p = 0.73; I^2^ = 49%, p = 0.09). The forest plot showed non-significant differences in HHS between the PRP group and the HA group at six months (SMD = 0.02, CI = -0.40, 0.44; p = 0.93; I^2^ = 64%, p = 0.06) and at 12 months (SMD = -0.31, CI: -0.32, 0.22, p = 0.73; I^2^ = 49%, p = 0.09).

Adverse Events and Complications

Out of the seven studies examined, four of them found no incidences of adverse effects or complications in patients who had intra-articular injection treatments with PRP or HA [21,22,24,26]. In three studies, patients reported experiencing post-injection pain which was consistently higher in the PRP group, of a temporary nature, and resolved on its own [20,23,25]. One study noted 10 pain reactions in the PRP group versus six in HA group [20]. In the second study, PRP participants experienced a significantly higher pain reaction than the HA group [23]. In the third study, there was a significantly higher incidence of pain during or after injection in the PRP group (3.50±2.22) versus the HA group (3.22±2.40) [25]. One study documented an isolated occurrence of a superficial hematoma that spontaneously resolved [20]. Another study noted temporary warmness, stiffness, and heaviness [25]. Table 3 outlines the adverse events and complications identified in the respective studies.

**Table 3:** Adverse events and complications of included studies.

Adverse event and complication records	Studies
No adverse events or complications with intra-articular injections for the PRP or HA groups	Dallari et al. [21], Di Sante et al. [22], Kraeutler et al. [24], Villanova-López et al. [26]
Pain during or after PRP or HA injection	Battaglia et al. [20], Doria et al. [23], Nouri et al. [25]
Superficial hematoma	Battaglia et al. [20]
Warmness, stiffness, and heaviness	Nouri et al. [25]

Discussion

The main findings of this systematic review and meta-analysis comparing the treatment of HOA symptoms with intra-articular injections of PRP versus HA were as follows: within one to two months of intra-articular injection, there were no differences in efficacy between PRP and HA for improving HOA pain; at six months, patients in the PRP group demonstrated significant improvements in pain and hip functional outcomes in comparison to patients treated with HA; in the long term, there were no differences between PRP and HA for improving pain and hip functional outcome, and; PRP and HA injections were mostly safe, however, PRP had higher pain adverse reactions, albeit self-limiting.

To the best of our knowledge, only a few systematic reviews and meta-analyses to date have compared the effectiveness of PRP and HA as treatments for HOA. Our study findings differed from the systematic review and meta-analysis by Belk et al. in the overall and mid-term evaluation of WOMAC and VAS scores [9]. While our study showed that PRP was significantly more beneficial for overall pain reduction, and pain improvement at six months, the study by Belk et al. showed no significant differences in the weighted improvement of any outcome score (WOMAC, VAS, or HHS) between PRP and HA groups at any time point [9]. For our primary outcome, it should be noted that our study had a larger sample size. Secondly, we utilized the WOMAC pain sub-scale for analysis instead of the complete WOMAC index. Thirdly, our study set a higher threshold for sub-analysis. Moreover, our study has evaluated adverse events as a secondary outcome, thus highlighting the safety profiles of these intra-articular injections.

Hip Pain

Pain is the main symptom for HOA patients which, along with stiffness, severely decreases the quality of life and joint function [1,27]. The underlying principle behind intra-articular administration is to alleviate pain by mimicking natural biological mechanisms within the hip joint. This approach aimed to address the limitations of other pharmacological and non-pharmacological interventions, which often result in inconsistent effectiveness, potential side effects, and an inability to influence the progression of the disease [28,29]. Intra-articular HA restores the viscoelastic properties of the synovial fluid, leading to a reduction of inflammation and functional improvement [30]. Intra-articular HA injection has emerged as a therapeutic option for HOA but hasn't been established as a gold standard for treatment [31]. Intra-articular injection of PRP, an autologous product rich in growth factors stored in platelet granules, stimulates chondrogenesis and reduces HOA catabolism and intra-articular inflammation [8,32]. PRP is generally considered safe, however, its recommendation for managing HOA remains uncertain due to a lack of evidence supporting its long-term benefits, and the absence of an established optimal PRP preparation and injection protocol [33,34].

In our study, no significant improvement in pain was noted between the PRP group and the HA group at one to two months. However, subsequent follow-up interventions at six months of the procedures differed among studies and the PRP group outperformed the HA group in terms of pain improvement. Some clinical trials evaluating intra-articular injections have posited that clinical effects were not present after the first intervention due to local swelling and pain at the puncturing point after injection which could influence the effect [10,35]. It is important to note that more studies were available for meta-analysis at the six-month time-point, which probably increased the chance of detecting a difference in measurement between the two groups. At 12 months, our analysis found no significant improvement in pain between the PRP group and the HA group. Moreover, Filardo et al. reported that positive clinical outcomes with PRP therapy are time-dependent, with an average efficacy duration of nine months, and that better and longer-lasting results are achievable in younger patients with mild HOA [36]. While they noted a trend toward better results with PRP between six and 12 months of follow-up in patients treated for knee OA, they did not demonstrate significantly improved results [10]. To the extent of our current understanding of the literature, very few clinical trials have evaluated therapeutic outcomes of PRP versus HA beyond one year [24].

Hip Dysfunction

The HHS was specifically devised to appraise the outcomes of hip impairments and treatment modalities among adults. It is a prominent clinician-based instrument that incorporates dimensions of pain, range of motion, and functional capacity [15,37]. The HHS is more responsive than generic tests such as the SF-36, tests of walking speed, and pain during walking, and is recommended for monitoring rehabilitation interventions focused on improving functional ability in patients with HOA [38]. Our study showed no significant difference in overall HHS between PRP and HA in all subgroups. We also found no significant differences in HHS between the PRP group and the HA group at six months and at 12 months. Given the chronic and slow progressing nature of HOA, longer follow-up time may be needed to detect reversal and functional improvements in the hip joint for patients treated with intra-articular injections. Similar findings were noted in previous studies evaluating HHS up to 12 months [9,31,39]. However, HHS was found to improve in studies evaluating PRP and HA independently [40,41].

Safety Profile of Intra-articular Injections

Our review demonstrated that post-injection pain was persistently more prominent in the PRP group in three studies [20,23,25]. This may have been influenced by PRP preparation techniques or needle choice. Studies by Filardo et al. demonstrated significantly more serious post-injection swelling and pain with intra-articular PRP injections compared to HA [10,35]. These pain reactions, however, were self-limiting, requiring no medical intervention [10]. Another study noted that pre-filling syringes prior to injection, standard aseptic protocol, proper needle choice, and isotonicity of the injected solution are techniques that may help to reduce the risk of adverse effects [42]. There was an isolated case of post-injection hematoma which may have arisen due to improper injection technique, and a few cases of warmness, stiffness, and heaviness after the injection which may have been attributable to the speed or volume of injection [20,25]. It is important to highlight that these occurrences were temporary and resolved without any lasting effects.

In more than 50% of the included studies, no patients reported adverse events or complications during or after the injections, which proved that both multiple PRP injections and HA injections administered over a long period of time were safe. Additionally, numerous reviews have also reached a similar consensus that PRP and HA injections are free of adverse events or complications [42,43]. Despite the remote risk of adverse events, intra-articular injections provide several advantages for addressing HOA including the ability to achieve high drug concentrations in the joint, limited bioavailability, and the reduction of adverse effects linked to overall circulation [42].

Limitations

Some limitations were present in this review that should be acknowledged. There was heterogeneity in the selected studies, primarily attributed to different PRP and HA preparations, dosing and injection intervals. In some cases, there were disparities in the treatment approach during the post-therapy period where some studies allowed concurrent pain and anti-inflammatory medications [21]. In other studies, anti-inflammatory drug usage was prohibited for time ranges between 48 hours and 16 weeks after treatment [20,22,24,25]. Secondly, the scales used to analyze pain and hip functional indexes differed across studies and this may have introduced some bias in the outcomes. Thirdly, there were a limited number of studies that had small sample sizes, thus underscoring the dearth of clinical research conducted on this specific topic to date.

This study, however, provided robust insight into the therapeutic potential and known challenges regarding the use of PRP for the treatment of HOA. Also, the standardized mean difference (SMD) was used as a summary statistic to account for the different psychometric scales used in evaluating the WOMAC score, VAS, and HHS across the studies, thus providing more standardized results.

Directions for Future Research

Future research designs employing a randomized double-blind methodology and inclusions of true control groups, such as sham treatments with saline, would eliminate some of the biases noted in our study. It is further recommended that the duration of total follow-up for future studies be prolonged beyond the first 12-month period. Moreover, in addition to patient-reported measures, the utilization of more objective tools, such as biomarkers or radiographic grading of HOA, to track the effectiveness of injection treatments during trials would provide an impartial measure of outcomes [44].

## Conclusions

The findings of this analysis indicate that PRP therapy may provide potential benefits and pose few risks for the treatment of HOA. PRP demonstrates a relatively greater effectiveness in relieving symptoms of HOA, particularly during the mid-term phase of a 12-month follow-up period. After six months of treatment, PRP exhibits notable improvements in pain reduction, surpassing the outcomes observed with HA treatment. During the initial two months of treatment and at the 12-month mark, no significant benefits of using PRP over HA are observed. Moreover, the administration of intra-articular injections of PRP and HA to patients with HOA has been demonstrated to be safe with no significant risks of major adverse events or long-term complications. Comprehensive, well-randomized controlled trials and larger sample studies with extended follow-up durations are essential for a thorough evaluation of the outcomes of PRP and HA injections before designating first-line intra-articular injection therapy for HOA.
